# Two-band and pauli-limiting effects on the upper critical field of 112-type iron pnictide superconductors

**DOI:** 10.1038/srep45943

**Published:** 2017-04-06

**Authors:** Xiangzhuo Xing, Wei Zhou, Jinhua Wang, Zengwei Zhu, Yufeng Zhang, Nan Zhou, Bin Qian, Xiaofeng Xu, Zhixiang Shi

**Affiliations:** 1 Department of Physics and Key Laboratory of MEMS of the Ministry of Education, Southeast University, Nanjing 211189, China; 2 Wuhan National High Magnetic Field Center, School of Physics, Huazhong University of Science and Technology, Wuhan 430074, China; 3 Advanced Functional Materials Lab and Department of Physics, Changshu Institute of Technology, Changshu 215500, China

## Abstract

The temperature dependence of upper critical field *μ*_*0*_*H*_c2_ of Ca_0.83_La_0.17_FeAs_2_ and Ca_0.8_La_0.2_Fe_0.98_Co_0.02_As_2_ single crystals are investigated by measuring the resistivity for the inter-plane (*H*//*c*) and in-plane (*H*//*ab*) directions in magnetic fields up to 60 T. It is found that *μ*_*0*_*H*_c2_(*T*) of both crystals for *H*//*c* presents a sublinear temperature dependence with decreasing temperature, whereas the curve of *μ*_*0*_*H*_c2_(*T*) for *H*//*ab* has a convex curvature and gradually tends to saturate at low temperatures. *μ*_*0*_*H*_c2_(*T*) in both crystals deviates from the conventional Werthamer-Helfand-Hohenberg (WHH) theoretical model without considering spin paramagnetic effect for *H*//*c* and *H*//*ab* directions. Detailed analyses show that the behavior of *μ*_*0*_*H*_c2_(*T*) in 112-type Iron-based superconductors (IBSs) is similar to that of most IBSs. Two-band model is required to fully reproduce the behavior of *μ*_*0*_*H*_c2_(*T*) for *H*//*c*, while the effect of spin paramagnetic effect is responsible for the behavior of *μ*_*0*_*H*_c2_(*T*) for *H*//*ab*.

Recently, the newly discovered 112-type Iron-based superconductors (IBSs) Ca_1−x_RE_x_FeAs_2_ (RE = rare-earth elements) have attracted tremendous interest due to their peculiar properties compared with other IBSs^1^,^2^. It crystallizes in a monoclinic crystal structure with a space group of *P*_21_, consisting of alternating stacking of FeAs and As zigzag bond layers. The FeAs layers have been proven to be responsible for the High-*T*_c_ superconductivity, while the unique As zigzag bond layers could generate anisotropic Dirac cones, and an additional three-dimensional (3 D) hole pocket which is absent in other IBSs^3^,^4^,^5^, and may also be possible for realizing the Majorana related physics^6^. However, so far the pair mechanism and many basic physical properties both in the superconducting and normal states are still not well understood in this compound. Upper critical field *μ*_*0*_*H*_c2_, is one of the most important superconducting parameters for gathering an understanding of unconventional superconductivity including coherence length, coupling strength, and insights into the pair-breaking mechanism. Moreover, the anisotropy of *μ*_*0*_*H*_c2_, which is related to the dimensionality and the topology of the underlying electronic structure, also becomes important for potential applications as well as for understanding multiband effects. Many efforts have been made to investigate the *μ*_*0*_*H*_c2_(*T*) ever since the discovery of IBSs^7^. For instance, in 1111-type IBSs, *μ*_*0*_*H*_c2,c_(*T*) shows a significant upturn behavior in (La, Nd)FeAs(O,F)^8^,^9^, and linearly increases with decreasing temperature but tends to be saturated at low temperatures in SmFeAs(O,F)^10^. Both behaviors can be accounted by the two-band theory^11^. *μ*_*0*_*H*_c2,ab_(*T*) exhibits a downturn and flattening curvature with decreasing temperature, which is mainly ascribed to the spin-paramagnetic effect^10^,^12^. For 122-type and 111-type IBSs, *μ*_*0*_*H*_c2,c_(*T*) exhibits a linear increase down to the lowest temperature, and *μ*_*0*_*H*_c2,ab_(*T*) also shows a downturn curvature with decreasing temperature. These behaviors can also be interpreted using the two-band theory and spin-paramagnetic effect, respectively^13^,^14^,^15^,^16^,^17^. Furthermore, spin-paramagnetic effect dominates in both of *μ*_*0*_*H*_c2,ab_(*T*) and *μ*_*0*_*H*_c2,c_(*T*) in the 11-type IBSs(e.g., Fe_1+y_Te_1−x_Se_x_)^18^,^19^. Nevertheless, the behavior of *μ*_*0*_*H*_c2_(*T*) at high fields and low temperatures is not clear yet in this newly discovered 112-type IBSs. Therefore, it is interesting to check whether Ca_1−x_RE_x_FeAs_2_ may represent a new type of IBSs, because the multiband electronic structure and peculiar properties in Ca_1−x_RE_x_FeAs_2_ may contribute to the behavior of upper critical filed.

Previous studies of *μ*_*0*_*H*_c2_(*T*) were just performed near *T*_c_ in low magnetic field^20^,^21^. Thus, measurement in higher magnetic field is essential to clarify the behavior of upper critical field and its anisotropy *γ* at low temperature region in this new type IBSs. Benefiting from the advanced technology of pulsed field measurement, in this work, we reported the temperature dependence of upper critical field *μ*_*0*_*H*_c2_(*T*) of Ca_0.83_La_0.17_FeAs_2_ and Ca_0.8_La_0.2_Fe_0.98_Co_0.02_As_2_ single crystals by measuring the electrical resistivity in pulsed fields up to 60 T at Wuhan National High Magnetic Field Center. The behavior of *μ*_*0*_*H*_c2_(*T*) and its anisotropy are systematically studied. Our results suggest that the two-band and spin-paramagnetic effects are shown to be responsible for the temperature-dependent behavior of *μ*_*0*_*H*_c2,c_(*T*) and *μ*_*0*_*H*_c2,ab_(*T*), respectively.

## Results

**Figure 1:**
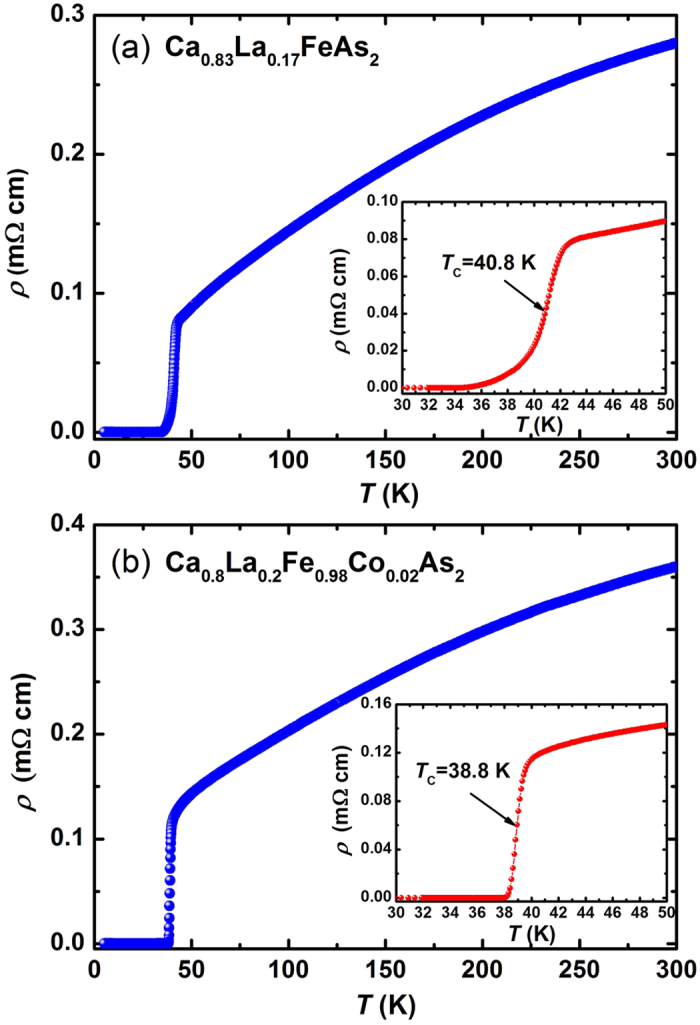
Temperature dependence of the in-plane electrical resistivity *ρ*(*T*) for (**a**) Ca_0.83_La_0.17_FeAs_2_ and (**b**) Ca_0.8_La_0.2_Fe_0.98_Co_0.02_As_2_ single crystals at zero field. The insets of (**a**) and (**b**) show an enlarged view of resistivity near the superconducting transition, *T*_c_ was determind by the 50% normal state resistivity *ρ*_n_.

Figure 1 presents the temperature dependence of the in-plane resistivity *ρ*(*T*) at zero field for (a) Ca_0.83_La_0.17_FeAs_2_ and (b) Ca_0.8_La_0.2_Fe_0.98_Co_0.02_As_2_ single crystals. The resistivity of both crystals monotonically decreases with decreasing temperature and shows no anomaly corresponding to the antiferromagnetic (AFM)/structural transition down to *T*_c_. The insets show the enlarged view near the superconducting transition. The transition temperature is estimated as 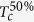 = 40.8 K for Ca_0.83_La_0.17_FeAs_2_ and 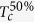 = 38.8 K for Ca_0.8_La_0.2_Fe_0.98_Co_0.02_As_2._ The transition width Δ*T*_c_, determined by adopting the criterion of 90%*ρ*_n_–10%*ρ*_n_, is 3.8 K for Ca_0.83_La_0.17_FeAs_2_, larger than the value of 1.1 K for Ca_0.8_La_0.2_Fe_0.98_Co_0.02_As_2_. The slightly wide superconducting transition for Ca_0.83_La_0.17_FeAs_2_ seems to be a general feature in this compound, which may result from the inhomogeneity of La distribution. Upon a small amount of Co doping, single crystal quality can be improved significantly with sharp superconducting transition and large superconducting volume fraction^20^,^21^,^22^,^23^.

**Figure 2:**
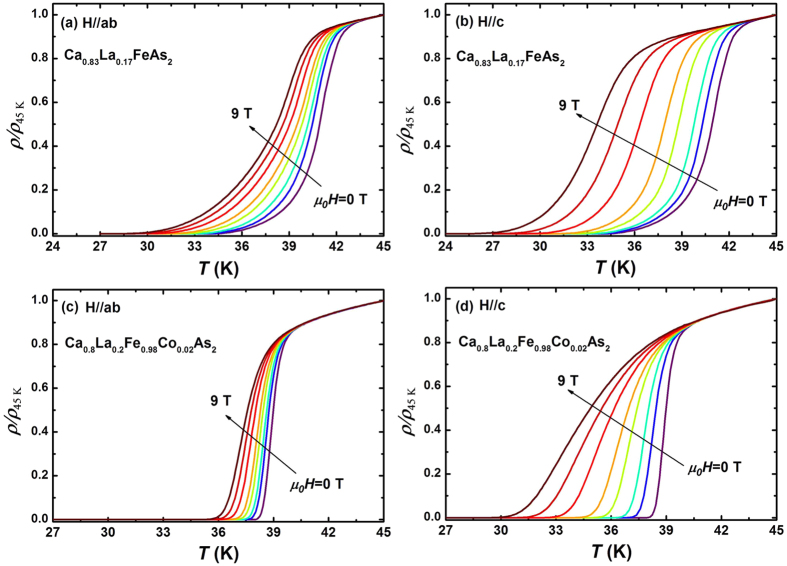
Temperature dependence of in-plane resistivity (nomalized to 45 K) of Ca_0.83_La_0.17_FeAs_2_ for (**a**) *H*//*ab* and (**b**) *H*//*c*, and of Ca_0.8_La_0.2_Fe_0.98_Co_0.02_As_2_ for (**c**) *H*//*ab* and (**d**) *H*//*c* at the various magnetic fields from 0 to 9 T(0, 0.5, 1, 2, 3, 5, 7 and 9 T).

Figure 2 shows the temperature dependence of the in-plane resistivity *ρ*(*T*) of Ca_0.83_La_0.17_FeAs_2_ and Ca_0.8_La_0.2_Fe_0.98_Co_0.02_As_2_ single crystals in low magnetic fields from 0 to 9 T for *H*//*ab* and *H*//*c*. With increasing fields, the resistivity transition width becomes broader and the onset of superconductivity shifts to lower temperatures gradually. It is worth noting that the superconducting transitions of Ca_0.83_La_0.17_FeAs_2_ and Ca_0.8_La_0.2_Fe_0.98_Co_0.02_As_2_ show different response to the increased magnetic field. For Ca_0.8_La_0.2_Fe_0.98_Co_0.02_As_2_, the field-induced broadening of superconducting transition is more pronounced for *H*//c, showing a tail structure at low temperatures, similar to the case in 1111-type IBSs and cuprates, which can be explained in terms of the formation of vortex-liquid phase^8^,^10^,^24^,^25^. In contrast, for Ca_0.83_La_0.17_FeAs_2_, the transition shows slightly field-induced broadening, indicating the vortex-liquid state region is very narrow, similar to the results of 122-type and 11-type IBSs^18^,^26^,^27^,^28^,^29^. This discrepancy seems to be consistent with the different vortex dynamics in both samples. Indeed, a small amount of Co doping distinctly influences the vortex dynamics and flux pinning, in comparison with Ca_0.83_La_0.17_FeAs_2_, the second magnetization peak was clearly observed and critical current density enhanced significantly through introducing more effective pinning center in Ca_0.8_La_0.2_Fe_0.98_Co_0.02_As_2_^23^,^30^.

**Figure 3:**
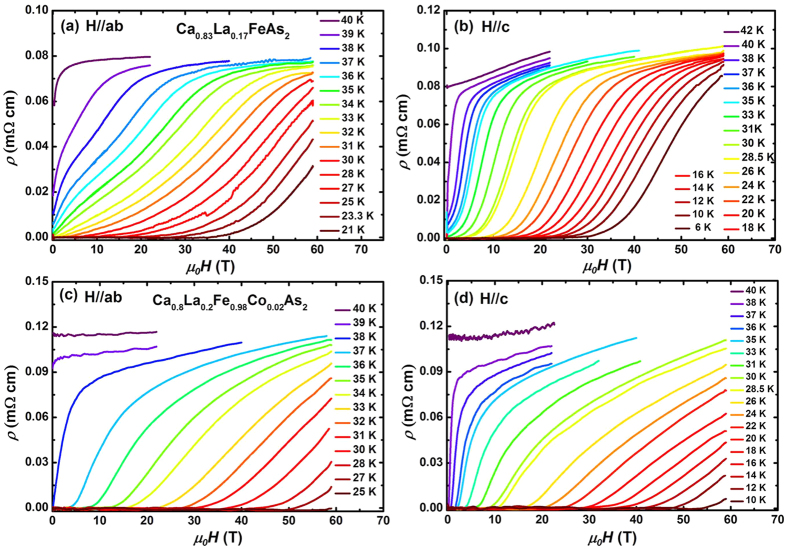
Magnetic field dependence of in-plane resistivity *ρ*(*H*) of Ca_0.83_La_0.17_FeAs_2_ for (**a**) *H*//ab and (**b**) *H*//c, and of Ca_0.8_La_0.2_Fe_0.98_Co_0.02_As_2_ for (**c**) *H*//ab and (**d**) *H*//c measured at fixed temperatures in pulsed fields up to 60 T.

The magnetic field dependence of resistivity *ρ*(*H*) of Ca_0.83_La_0.17_FeAs_2_ and Ca_0.8_La_0.2_Fe_0.98_Co_0.02_As_2_ single crystals for *H*//ab and *H*//c are presented in Fig. 3. Since there is no hysteresis for *ρ*(*H*) under *μ*_*0*_*H* sweep up and down processes, only data collected during the down sweep of the magnet are shown. Obviously, the superconducting transitions are suppressed upon increasing magnetic field for both directions. In addition, the superconductivity of Ca_0.8_La_0.2_Fe_0.98_Co_0.02_ is more robust against magnetic field compared with Ca_0.83_La_0.17_FeAs_2_, indicating Ca_0.8_La_0.2_Fe_0.98_Co_0.02_As_2_ has a higher *μ*_*0*_*H*_c2_(0) than Ca_0.83_La_0.17_FeAs_2_.

**Figure 4:**
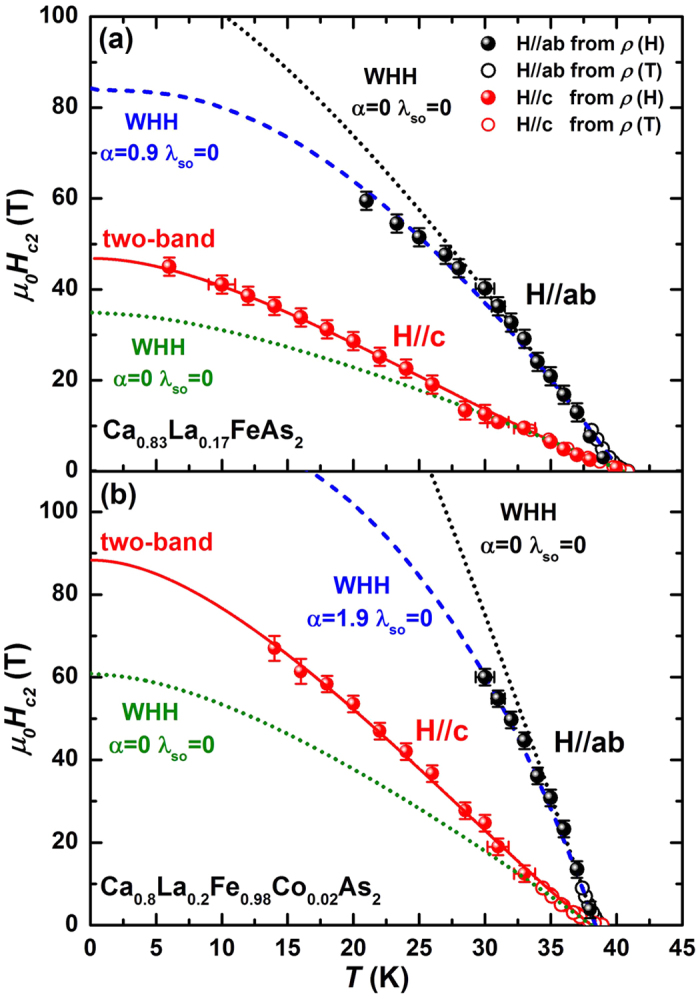
The upper critical field *μ*_*0*_*H*_c2_(*T*) versus temperature for (**a**) Ca_0.83_La_0.17_FeAs_2_ and (**b**) Ca_0.8_La_0.2_Fe_0.98_Co_0.02_As_2_ single crystals. Symbols of the open circles and filled circles represent the data obtained in the low magnetic field and pulsed field, respectively. The dotted lines are the WHH fits neglecting the spin paramagnetic effect for *H*//*ab* and *H*//*c*, respectively, while the dashed lines are the best fits to the experimental data by WHH model with the pauli-limiting effect considered for *H*//*ab*. The solid lines for *H*//*c* are fitting to the data using the two-band model.

In order to minimize the effects of superconducting fluctuation near 90%*ρ*_n_ and vortex motion in the vortex-liquid region near 10%*ρ*_n_ on the determination of *μ*_*0*_*H*_c2_, we use the 50%*ρ*_n_ criteria, which is widely accepted to be close to the real *μ*_*0*_*H*_c2_, to define the *μ*_*0*_*H*_c2_(*T*) values in the following^10^,^31^. The normal state resistivity *ρ*_n_ was determined by linearly extrapolating the normal state resistivity into the superconducting state in *ρ*(*T*) and *ρ*(*H*) curves separately. *μ*_*0*_*H*_c2_(*T*) of both crystals for *H*//*ab* and *H*//*c* directions along with the low magnetic field data up to 9 T were shown in Fig. 4. *μ*_*0*_*H*_c2_(*T*) obtained from the pulsed field measurement follows well the curvature and values of the low filed ones. Data above 60 T were extracted by linear extrapolation of *ρ*(*H*) at *μ*_*0*_*H* < 60 T to *ρ*(*H*) = 0.5*ρ*_n_(*T*_c_, *H*)^18^,^32^. In several highly two-dimensional superconductors, the curvature of *μ*_*0*_*H*_c2_(*T*) has been reported to vary depending on the criteria used to determine *μ*_*0*_*H*_c2_^8^,^14^. Thus, the *μ*_*0*_*H*_c2_(*T*) defined by 80%*ρ*_n_ and 20%*ρ*_n_ were also presented in Supplementary Information (SI), Fig. S2. It is noted that the shape of *μ*_*0*_*H*_c2_(*T*) curve does not change qualitatively when *μ*_*0*_*H*_c2_(*T*) is defined by different criteria in this compound. In addition, a slight upward behavior near *T*_c_ which commonly observed in some IBSs^14^,^33^,^34^, is also observed for both directions, might be due to the flux dynamics as is seen in cuprates^35^.

## Discussion

Generally, two distinct mechanisms exist in superconductivity suppression under magnetic fields in type-II superconductors. One is the orbital pair-breaking effect, with opposite momenta acting on the paired electrons. In this case, the superconductivity is destroyed when the kinetic energy of the Cooper pairs exceeds the condensation energy. The other is attributed to the spin-paramagnetic pair-breaking effect, which comes from the Zeeman splitting of spin singlet cooper pairs. The superconductivity is also eliminated when the Pauli spin susceptibility energy is larger than the condensation energy. WHH theory, which could identify the contribution of each pair-breaking mechanism, was used to fit the *μ*_*0*_*H*_c2_(*T*) curves, and the strength of the spin-paramagnetic effect and the spin-orbit effect were incorporated via the Maki parameter *α* and the spin-orbit interaction *λ*_so_, respectively^36^. According to the WHH theory, *μ*_*0*_*H*_c2_(*T*) in the dirty limit can be described by the digamma function^37^


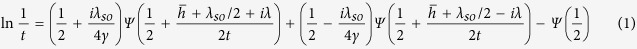


where 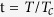, 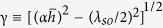 and 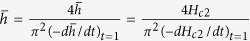.

In the absence of both spin-paramagnetic effect and spin-orbit interaction, *α* = 0 and *λ*_so_ = 0, the orbital-limited upper critical field is expressed as


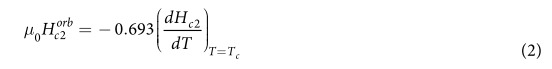


In the weakly coupled BCS superconductors, the pauli limited field is given by^38^


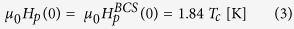


For conventional superconductors, *μ*_*0*_*Hp* (0) is usually much larger than *μ*_*0*_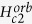 (0), and therefore, their upper critical field is mainly restricted by the orbital pair breaking mechanism. While, the spin-paramagnetic effect may play an import role in pair breaking in some unconventional superconductors^10^,^12^,^14^,^15^,^16^,^17^,^18^,^19^. In our case, we obtained 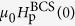 = 75 T for Ca_0.83_La_0.17_FeAs_2_ and 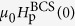 = 71.4 T for Ca_0.8_La_0.2_Fe_0.98_Co_0.02_As_2_. The slope values of *dμ*_0_*H*_c2_/*dT* near *T*_c_ is 3.98 T/K (9.22 T/K) and 1.23 T/K (2.26 T/K) for *H*//*ab* and *H*//*c* in Ca_0.83_La_0.17_FeAs_2_ (Ca_0.8_La_0.2_Fe_0.98_Co_0.02_As_2_), respectively. Using the WHH model, i.e. Eq. (2), one can estimate the orbital limited upper critical fields, which gives 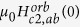 = 112.5 T (248 T) and 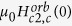 = 34.9 T (60.9 T) for Ca_0.83_La_0.17_FeAs_2_ (Ca_0.8_La_0.2_Fe_0.98_Co_0.02_As_2_). In both crystals, the values of 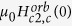 are much smaller than the corresponding *μ*_*0*_*H*^BCS^_p_(0). In contrast, 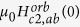 is much larger than 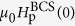. Thus, it is likely that the upper critical field in both crystals is limited by the orbital effect for *H*//*c*, but is limited by the spin paramagnetic for *H*//*ab*.

As evidenced from Fig. 4, the experimental *μ*_*0*_*H*_c2_(*T*) curves of both crystals deviate from the WHH model neglecting the spin paramagnetic effect (*α* = 0) and spin-orbit interaction (*λ*_so_ = 0) in *H*//*ab* and *H*//*c* at low temperatures (dotted lines). For *H*//*ab*, the curve of *μ*_*0*_*H*_c2_(*T*) falls below the WHH model (*α* = 0 and *λ*_so_ = 0) and has a tendency to saturate at low temperatures, indicating the spin-paramagnetic effect should be considered, as we discussed above. The best fits (dashed lines) were obtained using Eq. (1) with *α* = 0.9 for Ca_0.83_La_0.17_FeAs_2_, and *α* = 1.9 for Ca_0.8_La_0.2_Fe_0.98_Co_0.02_As_2_. It is noteworthy here that the spin-orbit scattering is not necessary to have the best fit (*λ*_so_ = 0). The negligible value of *λ*_so_ compared to the other IBSs indicates the spin-orbit scattering is also rather weak in this compound^10^,^12^,^16^,^18^,^32^. According to Maki^36^, the paramagnetic limited field 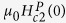 expresses as 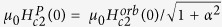, where the Maki parameter *α*, is given by 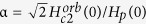, *μ*_*0*_*H*_p_(0) is the zero temperature pauli limited field. The values of *μ*_*0*_*H*_p,ab_(0) using *α* obtained from WHH model fitting for Ca_0.83_La_0.17_FeAs_2_ and Ca_0.8_La_0.2_Fe_0.98_Co_0.02_As_2_ are 176.75 T and 184.56 T, respectively, larger than the corresponding 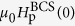 in both crystals. In IBSs, the weak-coupling BCS formula usually underestimates the actual paramagnetic limit. This enhancement of pauli limited field seems to be a common feature in IBSs, presumably aroused by many-body correlation and the strong coupling effects in IBSs^12^,^16^,^18^.

For *H*//*c, μ*_*0*_*H*_c2,c_(*T*) shows an almost linear temperature dependence and tends to be saturated at low temperatures, similar to the results of 1111-type SmFeAs(O,F) single crystals^10^. At low temperatures, the *μ*_*0*_*H*_c2,c_(*T*) is slightly larger than the value predicted with Eq. (2). The sublinear increase and enhancement of *μ*_*0*_*H*_c2,c_(*T*) are generally observed in many multiband superconductors, e.g., MgB_2_ and some IBSs^8^,^9^,^10^,^14^,^17^,^39^,^40^, which has been successfully explained by the two-band model^11^. The equation of *μ*_0_*H*_*c*2_(*T*) for a two-band superconductor is given by:


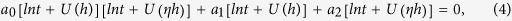


where t = *T*/*T*_*c*_, *a*_0_ = 2(*λ*_11_*λ*_22_ − *λ*_12_*λ*_21_)/*λ*_0_, *a*_1_ = 1 + (*λ*_11_ − *λ*_22_)/*λ*_0_, *a*_2_ = 1 − (*λ*_11_ − *λ*_22_)/1/2 *λ*_0_, *λ*_0_ = ((*λ*_11_ − *λ*_22_)^2^ + 4*λ*_12_*λ*_21_)^1/2^, *h* = *H*_c2_*D*_1_/(2*Φ*_0_/T), *η* = *D*_2_/*D*_1_ and *U*(x) = *Ψ*(1/2 + x) − *Ψ*(1/2). *Ψ*(x) is the digamma function, *D*_1_ and *D*_2_ are the diffusivity of each band, *λ*_11_, *λ*_22_ denote the intra-band coupling constants, and *λ*_12_, *λ*_21_ are the inter-band coupling constants. Due to the lack of microscopic theory of pairing mechanism, we choose the values of *λ* referring to previous reports in SmFeAs(O,F) from ref. 10. Here, we assume the intra-band coupling dominant the *μ*_0_*H*_c2,c_(*T*) and take the inter-band coupling value *λ*_12_ = *λ*_21_ = 0.17 (*λ*_12_ = *λ*_21_ = 0.13), and the intra-band coupling value *λ*_11_ = 0.7, *λ*_22_ = 0.3 (*λ*_11_ = 0.78, *λ*_22_ = 0.3) for Ca_0.83_La_0.17_FeAs_2_(Ca_0.8_La_0.2_Fe_0.98_Co_0.02_As_2_). The two-band model can fit the experimental data well as shown in Fig. 4 with *η* = 7.5 for Ca_0.83_La_0.17_FeAs_2_ and *η* = 8 for Ca_0.8_La_0.2_Fe_0.98_Co_0.02_As_2_. The values of *η* are close to that of SmFeAs(O,F) (*η* = 9)^10^. It should be noted that the results here are not sensitive to the choice of the coupling constants as discussed in previous report^9^,^14^,^41^. We find that the two-band model can also fit well, even if we change the value of *λ* properly. Although we can’t give the exact coupling values and enable us to analysis the possible pairing scenarios in this novel IBSs, the fitting results by two-gap model can be seen to agree very well with our experimental data.

In what follows, we discuss the different temperature dependence of *μ*_0_*H*_*c*2_(*T*) for *H*//*ab* and *H*//*c*, which was generally observed in IBSs^10^,^13^,^14^,^16^,^17^,^42^,^43^. Why the two-band model is essential to explain the behavior of *μ*_*0*_*H*_c2,c_(*T*), but the effect of spin paramagnetic effect is mainly responsible for the behavior of *μ*_*0*_*H*_c2,ab_(*T*), is still unclear now. This calls for further investigations on this interesting question, both experimentally and theoretically. Previous studies have proposed that the cross section of the Fermi surface produces closed current loops that form vortices for *H*//c due to the quasi-two-dimensional Fermi-surface. Thus, the orbital pair-breaking mechanism plays a dominant role in destroying the superconductivity in high magnetic fields, therefore, the two-gap theory, taking into account the orbital pair-breaking effect. In contrast, for *H*//ab, closed loops cannot be easily formed because the cross-sectional area of the Fermi surface is almost fully open with negligible orbital effect, thus, the spin paramagnetic effect is a more dominant factor in *μ*_0_*H*_*c*2*,ab*_(*T*)^10^,^17^. This scenario seems also to be suitable for this 112-type IBSs since all Fermi-surfaces exhibit two-dimensional character except for the *α* band^3^,^4^,^5^. Here, we try to give another possible scenario relevant to the spin-locked superconductivity, which has been proposed in quasi-one-dimensional superconductor K_2_Cr_3_As_3_^44^. The schematic diagram was shown in SI, Fig. S3. Under this physical scenario, the spins of Cooper pairs are predominantly aligned along the *ab*-plane in IBSs, thus, the behavior of *μ*_0_*H*_*c*2*,ab*_(*T*) would be pauli-limited. For *H*//*c*, since there is little spin along the c-axis, *μ*_0_*H*_*c*2*,c*_(*T*) would be hardly effected by the pauli pair-breaking, and mainly restricted by the orbital effect instead. In this case, due to the multiband electronic structure, the enhancement of upper critical field *μ*_0_*H*_*c*2*,c*_(*T*) should be described by the multiband theory model.

Superconducting parameters of Ca_0.83_La_0.17_FeAs_2_ and Ca_0.8_La_0.2_Fe_0.98_Co_0.02_As_2_ obtained from analysis above are summarized in Table 1. Using 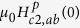 and 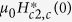, the superconducting coherence length *ξ* (0) can be estimated using the Ginzburg-Landau formula: *H*_c2,c_ = *Φ*_0_/2π 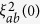 and *H*_c2,ab_ = *Φ*_0_/2π*ξab*(0) *ξ*_*c*_(0), where *Φ*_0_ = 2.07 × 10^−15^ T m^2^ is the flux quantum. We calculated *ξab*(0) = 2.65 nm and *ξ*_*c*_(0) = 1.47 nm for Ca_0.83_La_0.17_FeAs_2_, *ξab*(0) = 1.93 nm and *ξ*_*c*_(0) = 1.44 nm for Ca_0.8_La_0.2_Fe_0.98_Co_0.02_As_2_, respectively. *ξ*_*c*_(0) of both crystals is larger than the distance *d* (~1.035 nm) between the adjacent FeAs conducting layers, indicating a 3D superconductivity in this novel superconductor, despite the layered nature of their crystal structure.

**Figure 5:**
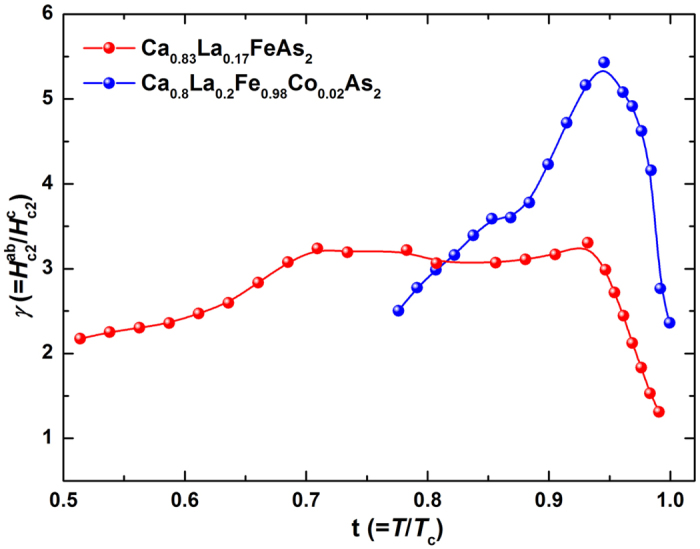
The anisotropic parameter *γ* versus the reduced temperature *T*/*T*_c_ for Ca_0.83_La_0.17_FeAs_2_ (red circles) and Ca_0.8_La_0.2_Fe_0.98_Co_0.02_As_2_ (blue circles) single crystals.

The temperature dependence of anisotropy of *μ*_*0*_*H*_c2_(*T*) is shown in Fig. 5 as a function of reduced temperature t = *T*/*T*_c_ for Ca_0.83_La_0.17_FeAs_2_ and Ca_0.8_La_0.2_Fe_0.98_Co_0.02_As_2_ single crystals. The values of *γ* reside in the range 1.2~3.2 (2.3 – 5.4) for Ca_0.83_La_0.17_FeAs_2_ (Ca_0.8_La_0.2_Fe_0.98_Co_0.02_As_2_) in the temperature region of *T* = (0.51 ~ 1) *T*_c_. The anisotropy *γ* values of both crystals show nonmonotonic temperature-dependent, which first increase and then decrease with decreasing temperature, in consistence with our earlier studies^23^. This strong temperature dependence of *γ* is similar to that of SmFeAs(O, F), which may originate from the combined effect of two-band nature and spin paramagnetism^10^. The decreasing *γ* with decreasing temperature in both crystals results from the enhanced *μ*_*0*_*H*_c2,c_(*T*) and the suppressed *μ*_*0*_*H*_c2,ab_(*T*).

In summary, we have investigated the temperature dependence of upper critical field of Ca_0.83_La_0.17_FeAs_2_ and Ca_0.8_La_0.2_Fe_0.98_Co_0.02_As_2_ single crystals under pulsed fields up to 60 T. Analysis based on the WHH model and two-band model indicates that, *μ*_*0*_*H*_c2_(*T*) of this compound bears many similarities to most of IBSs, *μ*_*0*_*H*_c2,ab_(*T*) is clearly limited by the pauli limited effect at low temperatures, and the two-band model is required to describe the enhancement of the upper critical field *μ*_*0*_*H*_c2,c_(*T*). Our work clearly clarifies the behavior of the upper critical field of 112-type iron pnictide superconductors.

## Method

### Single crystal growth and basic characterizations

Single crystals of Ca_0.83_La_0.17_FeAs_2_ and Ca_0.8_La_0.2_Fe_0.98_Co_0.02_As_2_ were grown using the flux method as previous reports^20^,^22^,^23^. The single crystal x-ray diffraction (XRD) was performed using a Rigaku diffractometer with Cu *K*α radiation (see SI, Fig. S1). Elemental analysis was performed by a scanning electron microscope equipped with an energy dispersive x-ray (EDX) spectroscopy probe.

### Electrical resistivity measurements

In-plane electrical resistivity was performed by the standard four-probe method in low magnetic field up to 9 T in a Quantum Design Physical Property Measurement System (PPMS-9T) and in pulsed field up to 60 T at Wuhan National High Magnetic Field Center. Golden contacts were made by sputtering in order to provide a low contact resistance (less than 1 Ω.) in the pulsed field measurement.

## Additional Information

**How to cite this article:** Xing, X. *et al*. Two-band and pauli-limiting effects on the upper critical field of 112-type iron pnictide superconductors. *Sci. Rep.* **7**, 45943; doi: 10.1038/srep45943 (2017).

**Publisher's note:** Springer Nature remains neutral with regard to jurisdictional claims in published maps and institutional affiliations.

## Supplementary Material

Supplementary Information

## Figures and Tables

**Table 1:** Superconducting parameters of Ca_0.83_La_0.17_FeAs_2_ and Ca_0.8_La_0.2_Fe_0.98_Co_0.02_As_2_ from the analysis of *μ*_*0*_*H*_c2_(*T*).

	*T*_c_ (K)	*μ*_*0*_ 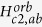 (0) (T)	*μ*_*0*_ 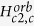 (0) (T)	*μ*_*0*_ 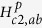 (0) (T)	*μ*_*0*_ 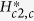 (0) (T)	*ξ*_*ab*_ (0) (nm)	*ξ*_*c*_ (0) (nm)
Ca_0.83_La_0.17_FeAs_2_	40.8	112.5	34.9	84.8	46.8	2.65	1.47
Ca_0.8_La_0.2_Fe_0.98_Co_0.02_As_2_	38.8	248	60.9	118.3	88.34	1.93	1.44

*μ*_*0*_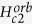 (0) denotes the orbital limited *μ*_*0*_*H*_c2_ at T = 0 K. *μ*_*0*_
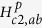
(0) denotes the paramagnetically limited *μ*_*0*_*H*_c2_ at zero-temperature for *H*//ab, which is estimated with the WHH theory including paramagnetism. *μ*_*0*_
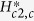
(0) is the upper critical field for *H*//c, which is determined from fittting the experimental data using the two-band model. *ξ*_ab_ (0) and *ξ*_c_ (0) are the *ab*-plane and *c*-axis zero temperature coherence length calculated using *μ*_*0*_
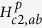
(0) and *μ*_*0*_
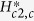
(0), respectively.
