# Supplementary material for: Two-band and pauli-limiting effects on the upper critical field of 112-type iron pnictide superconductors
Source: Sci Rep. 2017 Apr 6;7:45943. doi: 10.1038/srep45943 (PMC5382916; doi:10.1038/srep45943)
Supplement: Supplementary Information [file srep45943-s1.pdf]

Two-band and pauli-limiting effects on the upper critical field of 112-type iron pnictide superconductors

Xiangzhuo Xing[1], Wei Zhou[1], Jinhua Wang[2], Zengwei Zhu[2], Yufeng Zhang[1], Nan Zhou[1], Bin Qian[3,*], Xiaofeng Xu[3] & Zhixiang Shi[1,*]

[1]Department of Physics and Key Laboratory of MEMS of the Ministry of Education, Southeast University, Nanjing 211189, China

[2]Wuhan National High Magnetic Field Center, School of Physics, Huazhong University of Science and Technology, Wuhan 430074, China

[3]Advanced Functional Materials Lab and Department of Physics, Changshu Institute of Technology, Changshu 215500, China

Correspondence and requests for materials should be addressed to B. Q. (email: njqb@cslg.edu.cn) or Z. S. (email: zxshi@seu.edu.cn)

1. X-ray diffraction result

The single crystal x-ray diffraction (XRD) was performed using a Rigaku diffractometer with Cu *K*α radiation. Fig.S1. shows the single crystal XRD pattern of $Ca_{0.83}La_{0.17}FeAs_2$ and $Ca_{0.8}La_{0.2}Fe_{0.98}Co_{0.02}As_2$. It crystalized in a monoclinic crystal structure with a space group of P_{21}. Only (00*l*) diffraction peaks were observed, indicating good *c*-axis orientation.

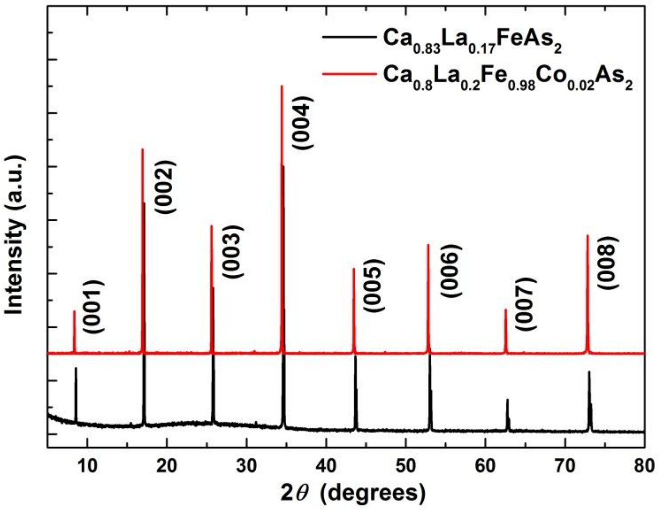

Fig. S1. X-ray diffraction pattern of $Ca_{0.83}La_{0.17}FeAs_2$ and $Ca_{0.8}La_{0.2}Fe_{0.98}Co_{0.02}As_2$ single crystals.

2. $\mu_0 H_{c2}$-T phase diagram

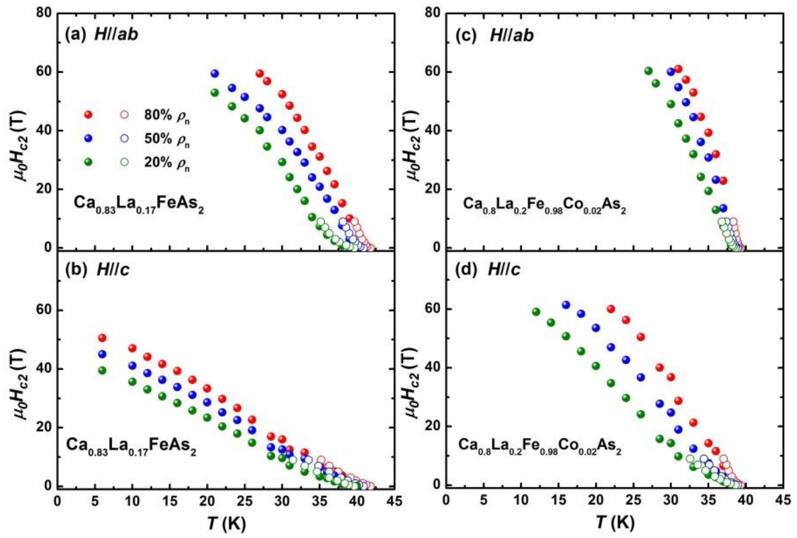

Fig. S2. Temperature dependence of the upper critical field $\mu_0 H_{c2}(T)$ of $Ca_{0.83}La_{0.17}FeAs_2$ for (a) $H//ab$ and (b) $H//c$, and of $Ca_{0.8}La_{0.2}Fe_{0.98}Co_{0.02}As_2$ for (c) $H//ab$ and (d) $H//c$ obtained from $\rho(T)$ (open symbols) and $\rho(H)$ (closed symbols) curves.

3. Schematic diagram

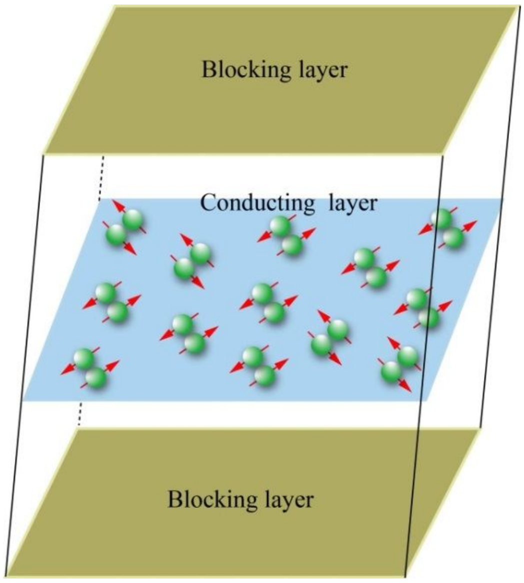

Fig.S3. The schematic diagram of spin-locked superconductivity in iron based superconductors. The blocking layer and conducting layer represent the CaAs layer and FeAs layer, respectively.
